# Weighted Gene Correlation Network Analysis (WGCNA) Reveals Novel Transcription Factors Associated With Bisphenol A Dose-Response

**DOI:** 10.3389/fgene.2018.00508

**Published:** 2018-11-12

**Authors:** Alexandra Maertens, Vy Tran, Andre Kleensang, Thomas Hartung

**Affiliations:** ^1^Center for Alternatives to Animal Testing, Bloomberg School of Public Health, Johns Hopkins University, Baltimore, MD, United States; ^2^Center for Alternatives to Animal Testing - Europe, University of Konstanz, Konstanz, Germany; ^3^Doerenkamp-Zbinden Professor and Chair for Evidence-Based Toxicology, Bloomberg School of Public Health, Johns Hopkins University, Baltimore, MD, United States

**Keywords:** bisphenol A, estrogen, WGCNA, ZNF217, TFAP2C, ZMYND8, PADI4, SREBF1

## Abstract

Despite Bisphenol-A (BPA) being subject to extensive study, a thorough understanding of molecular mechanism remains elusive. Here we show that using weighted gene correlation network analysis (WGCNA), which takes advantage of a graph theoretical approach to understanding correlations amongst genes and grouping genes into modules that typically have co-ordinated biological functions and regulatory mechanisms, that despite some commonality in altered genes, there is minimal overlap between BPA and estrogen in terms of network topology. We confirmed previous findings that ZNF217 and TFAP2C are involved in the estrogen pathway, and are implicated in BPA as well, although for BPA they appear to be active in the absence of canonical estrogen-receptor driven gene expression. Furthermore, our study suggested that PADI4 and RACK7/ZMYNDB8 may be involved in the overlap in gene expression between estradiol and BPA. Lastly, we demonstrated that even at low doses there are unique transcription factors that appear to be driving the biology of BPA, such as SREBF1. Overall, our data is consistent with other reports that BPA leads to subtle gene changes rather than profound aberrations of a conserved estrogen signaling (or other) pathways.

## Introduction

Bisphenol A (BPA) is an industrial chemical used in the manufacture of polycarbonate plastic found in a number of consumer products such as thermal paper, canned foods and epoxy resins (Rubin, 2011) – although many of these uses are being phased out (Zimmerman and Anastas, 2015). Amongst the general population, exposure to BPA is widespread, with very low levels of BPA present in the majority of urinary samples taken in the general population (Calafat et al., 2008), although serum levels are estimated to be lower (Teeguarden et al., 2013). Release of BPA to the environment exceeds one million pounds per year (Rubin, 2011).

Bisphenol-A has been subjected to a high-level of scrutiny – “bisphenol A” returns over 11,500 abstracts in PubMed, with over 700 articles per year being published every year since 2013 (“Pubmed Bisphenol A, n.d.). Within HSDB (the Hazardous Substance Database), there are over 79 peer-reviewed animal studies (TOXNET, n.d.). The CLARITY study, a three generation chronic study with low-levels of BPA used to mimic population exposures, involved 3,500 rats: while it resulted in no revision of safety standards by the FDA, it still failed to bring about a consensus as to a safe level (Academics Urge Caution in Interpreting Clarity-Bpa Results, n.d.). Despite the overwhelming amount of data, the mechanism(s) by which BPA may exert adverse effects remains unclear, nor is there a widely agreed upon endpoint on which to base a safe dose.

Bisphenol-A was presumed to have potentially estrogenic activity, as well as potential carcinogenicity, based on its structural similarity to DES (Diethylstilbestrol) and other synthetic estrogens, as well as appearing to trigger gene expression similar to estrogen receptor agonists, despite its relatively low binding affinity for estrogen receptors (LaPensee et al., 2009). Two different hypotheses have been put forward to explain this discrepancy: one, BPA may bind to different domains of ESR1 or ESR2 and recruit different co-regulators (Safe et al., 2002), or alternatively, BPA may exert its effects through non-classical estrogen receptors, such as membrane-bound ER (GPR30) (LaPensee et al., 2009) or ERRγ (ERRG) (Okada et al., 2008), which is one of several “orphan” receptors that are classified as estrogen-related receptors (Horard and Vanacker, 2003). The question of BPA’s ultimate molecular initiating event is not academic – on the presumption that BPA’s effects are mediated via estrogen receptors, several alternatives were proposed, such as Bisphenol F and Bisphenol S, but both compounds have proven equally problematic (Rochester and Bolden, 2015).

In our previous work for the Mapping the Human Toxome project (Kleensang et al., 2014; Bouhifd et al., 2015), we demonstrated that using non-inferential statistical methods that did not depend on existing annotations such as IDEA (Pendse et al., 2017) and WGCNA (Maertens et al., 2015) offered a powerful method to untangle possible regulatory mechanisms and providing insight into possible Pathways of Toxicity compared to either inferential-based methods or approaches such as pathway enrichment analysis that depend exclusively on annotations. Building upon our previous work using WGCNA applied to *in vitro* transcriptomic data to more fully understand the transcription factors that are driving the biology of estradiol (Pendse et al., 2017), we used WGCNA to examine a previously published transcriptomic dataset (Shioda et al., 2013). Briefly, Shioda et al. (2013) aimed to study the sensitivities of estrogen responsive genes to various endocrine disrupting chemicals (EDCs) based on the transcriptomic profile of MCF-7 cells exposed to either estrogen or several xenoestrogens (including BPA) over a dose-response curve ranging from picomolar to micromolar concentrations for a 48 h time period. Based on their analysis, they found that a gene signature of “estrogen-responsive genes” allowed the estrogenic substances to be ranked in terms of potency. Additionally, the heat map of BPA-inducible genes demonstrated a weak transcriptional activation at very low BPA concentration as well as a strong peak at high concentration. However, BPA has differences as well as similarities to estrogen in terms of gene signatures: therefore, we sought to explore specifically the differences between estrogen and BPA as well as the differences between low-dose BPA and high-dose BPA for possible regulatory mechanisms.

Our analysis shows that while there is substantial overlap between genes altered by BPA and estrogen, which might imply that BPA is indeed “estrogenic,” there are important differences in network topology as well as biological function, and that the overlap appears to be driven by transcription factors such as ZNF217, TFAP2C, PADI4, and RACK7/ZMYND8 rather than the estrogen receptor *per se*. Furthermore, BPA (even at the lower end of the dose response curve - defined here as less than 12.5 μM) has pathways that are likely not mediated by estrogen receptors, but instead by other transcription factors, such as SREBF1. Moreover, our data is consistent with other reports that BPA leads to subtle, diffuse gene changes that are comparatively difficult to capture with inferential methods, and that low-dose BPA has distinct effects compared to higher doses.

## Materials and Methods

### Data

Dataset GSE50705, a comprehensive analysis of estrogen and xenoestrogen dose-response curves on MCF-7 cells after 48 h of exposure, was downloaded from GEO via GEOQuery (Davis and Meltzer, 2007) as normalized data and all analyses were performed with R/Bioconductor (Gentleman et al., 2004).

### Weighted Gene Correlation Network Analysis

A WGCNA network (Langfelder and Horvath, 2007) was generated for several subsets of the data: Estrogen (*n* = 36), BPA (*n* = 44), and low dose BPA (*n* = 32), as well as a consensus network for estrogen and BPA together (*n* = 80) using the 10,000 most highly expressed genes for each subset of the data as determined by rank means expression, the approach in Maertens et al. (2015); consensus networks and module statistics followed overall the approach in Langfelder et al. (2008). Briefly, the network was derived based on a signed Spearman correlation using a β of 10 as a weight function. The topological overlap metric (TOM) (Yip and Horvath, 2007) was derived from the resulting adjacency matrix, and was used to cluster the modules using the blockwiseModules function (blockwiseConsensusModules, for the consensus modules) and the dynamic tree cut algorithm (Langfelder et al., 2008) with a height of 0.25 and a deep split level of 2, a reassign threshold of 0.2 and a minimum module size of 30 (100 for the consensus network). The eigenmodules— essentially the first principal component of the modules, which can be used as a “signature” of the modules gene expression —were then correlated with dose, and each module that was correlated with the dose-response curve with a *p*-value < 0.01 (*p*-value < 0.05 for the consensus network) was considered statistically significant.

### Transcription Factor Analysis

All statistically significant modules were analyzed in EnrichR (Chen et al., 2013) using the CHEA dataset (Lachmann et al., 2010) restricted to MCF-7/10 cells –as well as the ARCHS4 TF-Coexpression dataset with an adjusted *p*-value less than 0.01 based on Fisher’s exact test.

### Functional Annotation Analysis

Module “hubs” were defined as having high-ranking kME (which ranks the connectivity of genes) within the module and predicted as high-degree within the STRING database (Szklarczyk et al., 2017) of protein–protein interactions. The three highest ranking modules were analyzed in STRING for the enrichment of predicted protein interactions as well as functional annotation via GO Biological Process and Molecular Function. All analysis with STRING was done with medium stringency settings, and included all possible interactions (text-mining, database, experiments, co-expression, neighborhood, gene fusion, and co-occurrence).

### TCGA Data

Expression and methylation data for FIZ1 was correlated with clinical attributes (estrogen receptor, progesterone receptor, and solid tumor vs. normal vs. metastatic tumor) using MEExpress (Koch et al., 2015) based on the TCGA BRCA dataset.

## Results

### Consensus Network Analysis Indicates Minimal Overlap Between Estrogen and BPA

**FIGURE 1:**
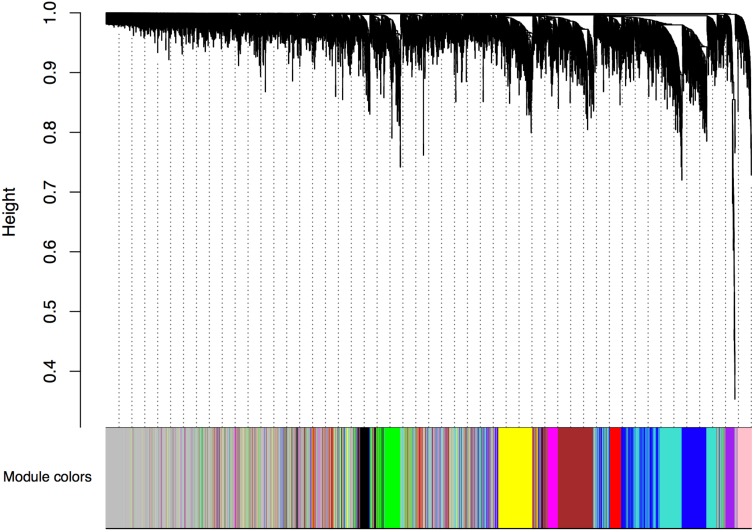
Consensus network from BPA and Estrogen dose-response curve. Gene expression similarity is determined using a pair-wise weighted correlation metric, and clustered according to a topological overlap metric into modules; assigned modules are colored on bottom, gray genes are unassigned to a module.

**FIGURE 2:**
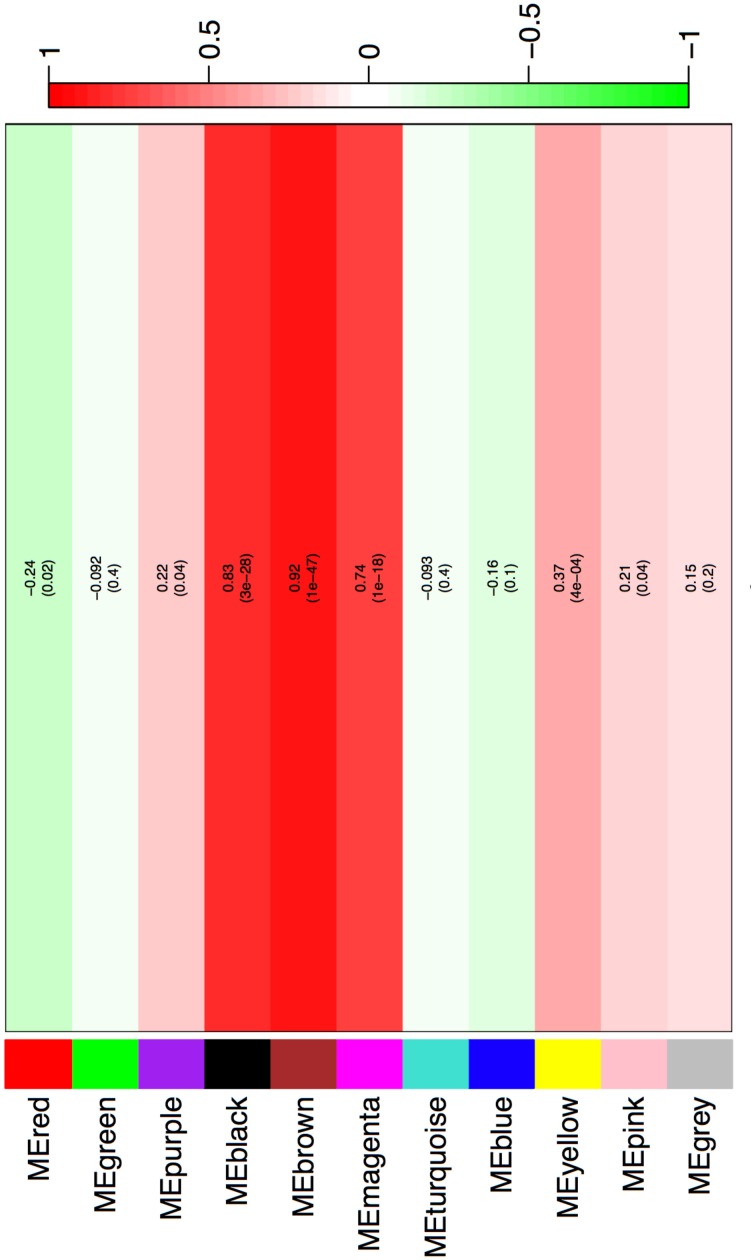
Consensus network modules correlated with estrogen dose using the eigenmodule (the first principal component of the module). Correlation coefficient along with *p*-value in parenthesis underneath; color-coded according to correlation coefficient (legend at right).

**FIGURE 3:**
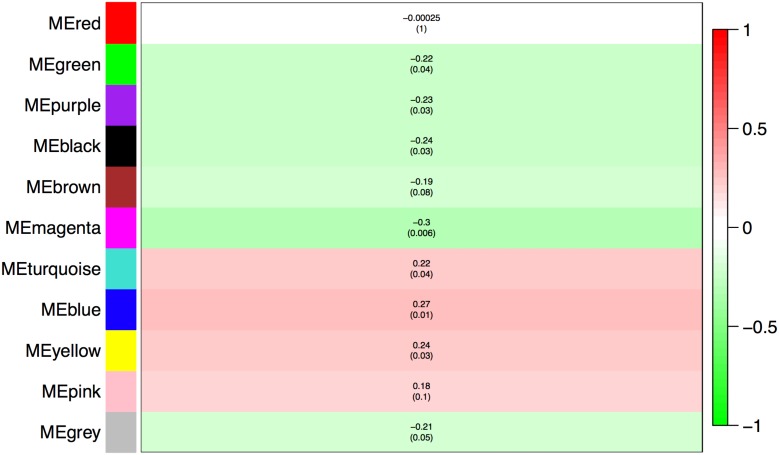
Consensus network modules correlated with BPA dose using the eigenmodule (the first principal component of the module). Correlation coefficient along with *p*-value in parenthesis underneath; color-coded according to correlation coefficient (legend at right).

We began by analyzing the dose-response curve of the BPA and estrogen dataset combined using WGCNA (which takes advantage of correlations amongst genes and groups genes into modules using network topology) to look for a “consensus network”-a common pattern of genes that are correlated in all conditions. The consensus network identified (Figure 1) had clearly delineated modules, and the modules identified were significantly correlated with both estrogen (Figure 2) and BPA (Figure 3). However, estrogen clearly had a stronger signal in comparison to BPA and quite possibly overwhelmed the signal from BPA. More strikingly, however, the majority of modules in the consensus analysis when analyzed for correlation with both BPA and estrogen showed virtually no similarity – most modules had opposite directions of correlation, and of the few modules with similar correlations, the coefficient of correlation was very weak – only one module (“yellow”) was significant with a *p*-value < 0.05 (Table 1), indicating that while there may be some overlap in genetic signatures, from a network topology perspective there is minimal conservation. When analyzed for transcription factors against the CHEA dataset – a collection of ChIP-chip, ChIP-seq, ChIP-PET, and DamID studies collected into a database to infer transcriptional regulation (Lachmann et al., 2010) – the common module was enriched for E2F1, ZNF217, and RACK7, but not ESR1 or ESR2 (Table 2).

**Table 1:** Consensus network modules associated with BPA and estrogen.

Module	Correlation	*p*-value
Red	-0.00025	1
Green	-0.092	0.4
Purple	NA	NA
Black	NA	NA
Brown	NA	NA
Magenta	NA	NA
Blue	NA	NA
Yellow	0.24	0.03
Pink	0.18	0.1
Gray	NA	NA

*Consensus network modules were correlated against both estrogen and BPA; NA indicates that the modules had different directions of correlation in estrogen compared to BPA.*

**Table 2:** Enriched transcription factors in conserved module in consensus network.

Transcription factor	Adjusted *p*-value
E2F1	0.000003501
ZNF217	0.000004066
RACK7/ZMYNDB	0.005142

*Transcription factors significantly enriched in the conserved module (“yellow”) between BPA and estrogen.*

### Estrogen and BPA Network Overlap With Transcription Factors, Including ESR1 and ESR2, but With Different Network Topologies and Different Biological Processes

Next, we derived the *de novo* networks individually for the entire dose-response curve of estrogen and BPA to examine the network topology, common hubs, and biological role of the modules in each network separately. Within the estrogen network (Supplementary Figure 1A), there was one large module highly correlated with estrogen dose (“turquoise”) (Table 3), which was also enriched for ESR1 and ESR2 in addition to E2F1, ZNF217, TFAP2C amongst others (Table 4); furthermore, ESR1 was a hub within the module, and the module was predominately enriched with terms related to cell-cycle as well as poly(A) RNA binding (Supplementary Table 1).

**Table 3:** Estrogen modules correlated with dose.

Module	Correlation	*p*-value
Turquoise	0.710543783	1.73E-06
Dark Green	0.667630497	1.18E-05
Dark Red	0.548734271	6.42E-04
Light Yellow	0.470241047	4.36E-03
Brown	0.45994764	5.44E-03
Salmon	0.443256223	7.66E-03
Gray60	-0.47528565	3.91E-03
Blue	-0.5389571	8.36E-04
Yellow	-0.55352085	5.62E-04
Black	-0.63950993	3.54E-05
Midnight Blue	-0.73602275	4.69E-07

*All modules correlated with estrogen dose-response curve with a p-value less than 0.01.*

**Table 4:** Enriched transcription factors in estrogen modules.

Module	TF	Adjusted *p*-value
Black	RACK7	0.005152
Blue	ZNF217	2.79E-14
	PADI4	1.10E-08
	RACK7	8.23E-07
	TFAP2C	0.00001662
	GATA3	0.00004515
	FOXM1	0.000373
	E2F1	0.000674
Turquoise	E2F1	1.43E-20
	ESR1	7.21E-10
	ESR2	2.12E-09
	PADI4	1.34E-07
	RACK7	7.39E-07
	GATA3	0.0000105
	RUNX1	0.0003015
	ZNF217	0.0006679
	TFAP2C	0.0008849
	ELK1	0.001171
	FOXM1	0.001239
Yellow	PADI4	0.001012
	RUNX1	0.001601

*All statistically significant modules correlated with dose, with enriched transcription factors. Modules that did not have TFs with an adjusted p-value ≤ 0.01 were excluded (Dark Red, Dark Green, Salmon, Midnight Blue, Gray60).*

In comparison, the top module correlated with BPA dose (“lightcyan1”) (Supplementary Figure 1B) (Table 5), was a relatively small module not enriched for any transcription factors, however, SSR1 (Signal Sequence Receptor Subunit 1) was the top hub; annotation analysis revealed that the module was enriched for genes in the GO category of “response to endoplasmic reticulum stress” and “endoplasmic reticulum unfolded protein response” (*p*-value of 2.93E-17 and 2.02E-12, respectively) (Supplementary Table 1). The second module correlated with dose (“royal blue”) was enriched for ESR1 and ESR2 genes (Table 6), however, neither ESR1 nor ESR2 was present in the module (or any module correlated positively or negatively with dose) and instead the main hub was TOP2A (Topoisomerase IIA). The module had a weak over-representation of genes involved in development (*p*-value 0.00416) and cytoskeleton organization (*p*-value 0.0155) (Supplementary Table 1). The other module enriched for ESR1 and ESR2 genes (“dark gray”) was also annotated to “response to unfolded protein” (*p*-value of 9.52E-05) (Supplementary Table 1).

**Table 5:** BPA modules associated with dose.

Module	Correlation	*p*-Value
Lightcyan 1	0.725175961	6.17E-15
Royal Blue	0.423995959	5.84E-05
Dark Gray	0.390795349	2.38E-04
Light Yellow	0.383058412	3.23E-04
Ivory	-0.372076037	4.92E-04
Light Cyan	-0.291083662	7.23E-03
Green	-0.280875808	9.65E-03
Gray60	-0.279474408	1.00E-02

*All BPA modules correlated with estrogen dose-response curve with a p-value less than 0.01.*

**Table 6:** Enriched transcription factors in BPA modules.

Module	TF	Adjusted *p*-value
Dark Gray	ESR1	1.32E-16
	ESR2	2.74E-08
	ZNF217	0.000002163
	GATA3	0.00001401
Green	ZNF217	1.59E-11
	RACK7	0.00006931
	ESR2	0.0001032
	GATA3	0.0002586
	TFAP2C	0.00055
	ESR1	0.002135
	PADI4	0.002604
	FOXM1	0.003341
Light Cyan	TFAP2C	0.002078
	GATA3	0.002583
Royal Blue	ZNF217	5.24E-08
	ESR2	7.76E-07
	ESR1	0.000005588
	ARNT	0.00005395
	AHR	0.0002705
	GATA3	0.002178

*All statistically significant modules correlated with dose, with enriched transcription factors. Modules that did not have TFs with adjusted p-value ≤ 0.01 were excluded (LightCyan1, Ivory, Light Yellow, Plum, Gray60).*

#### Low-Dose BPA Network Shows No Enrichment of ESR1 or ESR2 Genes

It has been speculated that BPA at low doses has fundamentally different effects than at high doses; in the original study of the dataset, the authors detected a weak, but distinct, transcriptional activity peak at low doses. Therefore, we restricted the BPA network to doses below 12.5 μM (leaving a highest dose of 6.25 μM, and most of the dose-response curve in the nanomolar/picomolar range) and calculated a network specific for this lower dose range. Despite the smaller sample size, the network still produced several modules that were significantly correlated with dose (Supplementary Figure 1C and Table 7). This low-dose BPA network shows consistent transcription factors (ZNF217, TFAP2C, RACK7/ZMYND8, and PADI4) with the larger BPA network as well as the estrogen network, but no modules were enriched for genes with ESR1 or ESR2 with a *p*-value cut-off of < 0.01 (Table 8).

**Table 7:** Low-dose BPA modules associated with dose.

Module	Correlation	*p*-value
Turquoise	0.71054	5.21E-06
Dark Green	0.66763	2.99E-05
Dark Red	0.54873	1.15E-03
Light Yellow	0.47024	6.61E-03
Brown	0.45995	8.08E-03
Salmon	0.44326	1.11E-02
Grey60	-0.4753	5.98E-03
Blue	-0.539	1.46E-03
Yellow	-0.5535	1.02E-03
Black	-0.6395	8.13E-05
Midnight Blue	-0.736	1.58E-06

*All low-dose BPA modules correlated with estrogen dose-response curve with a p-value less than 0.01.*

**Table 8:** Enriched transcription factors in low-dose BPA modules.

Module	TF	Adjusted *p*-value
Black	RACK7	1.78E-07
	TFAP2C	0.00001339
	RUNX1	0.00004104
Blue	ELK1	0.00000437
	ZNF217	6.14E-07
	PADI4	0.000007639
	FOXM1	9.66E-08
	HIF1A	0.005915
	AHR	0.00208
	E2F1	0.002221
	ARNT	0.005915
	RUNX1	0.008124
	GATA3	0.008124
Brown	E2F1	0.002459
	PADI4	0.006908
Turquoise	ZNF217	0.00000179
	RACK7	0.00001912
	GATA3	0.00002574
	PADI4	0.0001018
	FOXM1	0.0001703
	RUNX1	0.0004958
	E2F1	0.001544
Yellow	E2F1	6.34E-18
	PADI4	0.003362
	RACK7	0.00334
	FOXM1	0.009047

*All statistically significant modules correlated with dose, with enriched transcription factors. Modules that did not have TFs with adjusted p-value ≤ 0.01 were excluded (Light Yellow, Salmon, Gray60, Dark Green, Midnight Blue, Dark Red).*

The module with the highest correlation with dose (“turquoise”) was comparatively dense for predicted protein-protein interactions (*p*-value of < 1.0e-16, average node degree 10.3) as well as genes related to cellular macromolecule metabolic process and poly(A) RNA binding (*p*-value of 1.58E-16 and 2.17eE-18, respectively) (Supplementary Table 1) in contradistinction to the estrogen network, where the dominant module was enriched overwhelmingly with cell-cycle genes. Moreover, the module included both ZNF217 and TFAP2C, but neither ESR1 nor ESR2 were in the module, much less hubs. The second module correlated with dose (“Dark Green”) showed no enrichment for transcription factors, although it was enriched for protein–protein interactions and the molecular function “enzyme binding”; the third module (“dark red”), also strongly correlated with dose, showed no enrichment for transcription factors or protein–protein interactions, though it was weakly enriched for the KEGG pathway Insulin Signaling (*p*-value 0.0028); this module may simply be an artifact, reflect diffuse alterations that are difficult to detect, or an unknown regulatory mechanism. An additional fairly large module correlated with dose (“brown”) was enriched for both E2F1 and PADI4, strongly enriched for protein-protein interactions (*p*-value < 1.0E-16, average node degree 6.56) and as well as cellular metabolic process (*p*-value 1.32E-10) and poly(A) RNA binding (*p*-value 2.5E-16) (Supplementary Table 1); but, also in contrast to the estrogen network, was not enriched for cell-cycle genes.

ZNF217 has previously been shown by our work (Pendse et al., 2017) and others (Frietze et al., 2014) to be a critical component of estrogen signaling and an important prognostic factor for breast cancer (Vendrell et al., 2012). Similarly, TFAP2C is known to modulate ESR1 and GPR30 expression, and attenuate the expression of several estrogen-targeted genes (Woodfield et al., 2007). Given the presence of both ZNF217 and TFAP2C in the network as well as the strong enrichment genes targeted by these transcription factors, this suggests that these genes are indeed central to mediating BPAs phenotypic effects; however, our study shows little evidence that they are in exerting their effect in tandem with ESR1 or ESR2.

Moreover, both ZNF217 and TFAP2C were shown independently to be altered by bisphenol A in a rat seminiferous tubule culture model (Ali et al., 2014). The same study also showed alterations (albeit subtle) in PADI4 and RACK7 (official gene symbol: ZYMND8) mRNA as well as RACK7/ZMYND8 methylation; neither of these genes were in our dataset so their role in observed changes remains speculative. However, RACK7/ZYMND8 binds a large set of active enhancers, including almost all “super-enhancers,” and is therefore expected to have sweeping transcriptional effects (Shen et al., 2016). Although little is known about its role in breast cancer, it is thought to inhibit HIF-dependent breast-cancer progression (Chen et al., 2018). PADI4 is known to be implicated in cancer and is thought to respond to estrogen-simulation in MCF-7 cells through both genomic and non-genomic mechanisms (Dong et al., 2007). In breast cancer specifically, it is implicated in the ELK1/C-Fos pathway (Zhang et al., 2011). Moreover, BPA was shown to increase protein levels of PADI4 via a reactive oxygen species mechanism in neuroblastoma cells (Park et al., 2012).

### Low-Dose BPA Network Had Unique Transcription Factors Not Present in the Estrogen Dataset

To further delineate possible transcription factors unique to BPA signaling compared to estrogen, we examined the list of genes in all modules statistically significantly associated with the low-dose BPA network that were not present in the estrogen network, a total of 1,901 genes. Analyzed against the CHEA dataset, the genes were again enriched for RACK7/ZMYND8, in addition to ELK1 and HIF1A (Supplementary Table 2). In order to expand our search for transcription factors that may not have been studied in MCF-7 cells in the CHEA data set, we also analyzed the list of genes for enrichment against the ARCHS4 database (Lachmann et al., 2018), which correlates transcription factor expression against gene expression in a combined database of over 20,000 RNASeq samples. Of the top 50 transcription factors identified as significantly correlated with the gene list, 18 were also present in the low-dose BPA network (Table 9). The highest-ranking transcription factor, FIZ1, is zinc-finger protein with a largely unknown biological role (Wolf and Rohrschneider, 1999) - it has a relatively poor literature base, with only 8 citations in PubMed. However, FIZ1 expression in breast cancer is statistically associated with progesterone receptor status, estrogen receptor status, and sample subtype, and it undergoes extensive CpG-island methylation (Supplementary Figure 2), and it is therefore an intriguing candidate for further study. The second highest-ranking transcription factor, SREBF1 is comparatively better characterized: it is known to be central to lipid homeostasis, regulating the LDL receptor gene as well as related fatty acid and cholesterol synthesis genes. Furthermore, SREBF1 mRNA was identified as upregulated in adipocytes by BPA (Boucher et al., 2014). Neither SREBF1 nor FIZ1 were present in the estrogen dataset, and SREBF1- and FIZ1-correlated genes were not enriched in the subset of estrogen-only genes. It is therefore plausible that these two transcription factors are more central to BPAs effects than estrogen, however, because enrichment for transcription factors motifs/regulated genes in any gene list often produce false-positives, understanding their role would require further study.

**Table 9:** Transcription factors unique to low-dose BPA network.

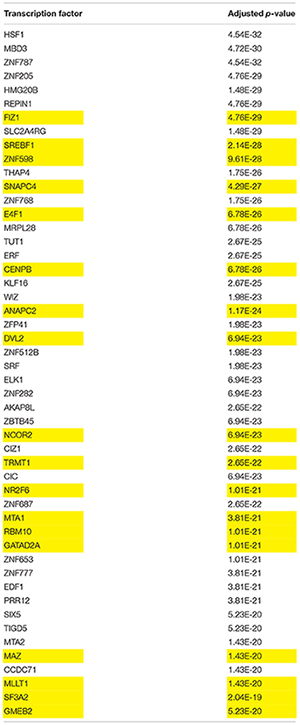

*Genes unique to low-dose BPA compared to estrogen were analyzed against the ARCHS4 for potential transcription factor enrichment; all the transcription factors also present in the dataset were identified in yellow.*

## Discussion

Estrogen signaling is unique amongst nuclear receptors in that substantial number of the genes altered by estrogen do not have canonical estrogen response elements (Miller et al., 2017) – estrogen signaling takes place within a transcriptomic and epigenomic context that markedly influences receptor activation. Our examination of the estrogen dose response curve network both confirmed several of the transcription factors identified previously, such as E2F1, ZNF217 and TFAP2C, as well as suggested other transcriptional factors such as PADI4 and RACK7/ZYMND8 that may impact estrogen signaling.

Our study is consistent with other findings that the assumption that BPA works exclusively or even predominantly on canonical ESR1 or ESR2 gene regulation may be misleading or an oversimplification (Delfosse et al., 2012; MacKay and Abizaid, 2018). To be sure, one can find gene patterns similar to those found in estrogen-induced cells, but the leap from that observation to the presumption that such changes are estrogen-mediated may not be warranted. While this study cannot determine conclusively the ultimate chain of events that leads from the molecular initiating event to the phenotypic consequences, it does suggest some hypotheses that are more probable. The lack of overlap in the consensus network indicates that despite similarity of genes, there is minimal conservation of network topology, and the one conserved module was not enriched for ESR1 or ESR2 genes. In networks drawn separately from dose-response curves for estrogen and BPA, the substantial differences in network topology, the absence of ESR1 as a hub gene in the BPA network, and the differences in biological function of the modules suggest that even at high-doses, BPAs effects are fundamentally different than estradiol. The lack of estrogen receptor target genes in the low dose BPA network in the presence of a clear signature of other transcription factors suggests that at low doses BPA’s effects are driven by mechanisms other than direct estrogen receptor activation. Additionally, regardless of molecular initiating event, assessing BPAs dose-response by looking at estrogen gene-signatures may miss interesting and important biology, such as the likely role of SREBF1. Furthermore, our study is consistent with other findings that BPA’s effects are subtle and phenotypic changes likely reflect modest effects at multiple different points (Porreca et al., 2016) and that analyzing the effects of low-dose BPA can reveal effects that are obscured at higher doses (Shioda et al., 2013). This does not necessarily lead to a “non-monotonic” dose response curve– this could be due to technical reasons, or higher-doses could cause non-specific changes that are the result of cellular stress, as evidenced by the identification of modules associated with unfolded protein response. It does, however, point to a need to consider the doses chosen for an *in vitro* study carefully and to not presume linear effects.

This study is certainly not a definitive study of BPA molecular mechanisms: our conclusions cannot confidently be extrapolated to other tissue types, as BPA may have tissue specific effects; MCF-7 cells are prone to artifacts (Kleensang et al., 2016); and our study did not focus on epigenetic mechanisms which are speculated as significantly underpinning much of the observed adverse events seen with BPA exposure, especially at a low dose (Singh and Li, 2012). While using the CHEA dataset and restricting candidate transcription factors to those observed in MCF-7 cells eliminates many of the false-positives intrinsic to such approaches, it also limits findings to those transcription factors that have been studied, and this may miss some important biology. Extending our analysis with the ARCHS4 database added interesting candidates, but all correlation-based approaches must be treated with caution and viewed as “hypothesis-generating,” and all exploratory data analysis techniques such as WGCNA require further targeted studies to confirm suggested molecular networks.

Nonetheless, our study does indicate that transcriptomics, especially given a high-dimensional dataset and the use of non-inferential methods, can likely aid toxicologists in having a better understanding of probable molecular targets as well as the complexity of perturbed networks - clearly, understanding BPAs effects will require a systems level approach (Hartung et al., 2017) as well as better characterization of genes that are not as yet confidently mapped as to biological function. More generally speaking, this points to the pitfall of trying to design “greener” substitutes (Maertens et al., 2014; Maertens and Hartung, 2018) in the absence of a clear, comprehensive understanding of molecular mechanism.

## Author Contributions

AM: main author of the paper. VT: support programming and data analysis. AK: planned the work and revised the manuscript. TH: mentoring, revision of the manuscript, and PI Human Toxome project laying the conceptual ground.

## Conflict of Interest Statement

The authors declare that the research was conducted in the absence of any commercial or financial relationships that could be construed as a potential conflict of interest.

## Supplementary Material

FIGURE S1 Estrogen and **(B)** BPA Network dendrogram.

FIGURE S2 Methylation pattern for FIZ1 in BRCA TCGA data set.

TABLE S1 Protein-protein interaction and enrichment from STRING database.

TABLE S2 Transcription factors for genes present only in low-dose BPA network.
