# Supplementary material for: Weighted Gene Correlation Network Analysis (WGCNA) Reveals Novel Transcription Factors Associated With Bisphenol A Dose-Response
Source: Front Genet. 2018 Nov 12;9:508. doi: 10.3389/fgene.2018.00508 (PMC6240694; doi:10.3389/fgene.2018.00508)
Supplement: FIGURE S1 — Estrogen and (B) BPA Network dendrogram. [file Data_Sheet_1.PDF]

Supplemental Figure 1

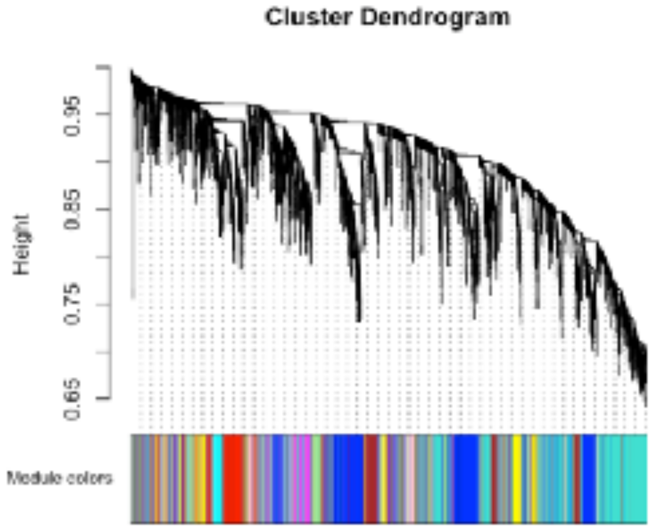

1A: ESTROGEN NETWORK DENDROGRAM

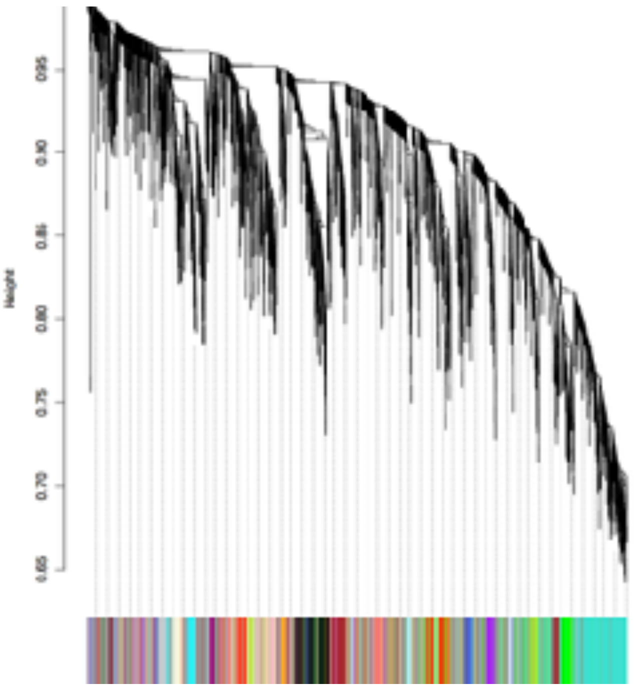

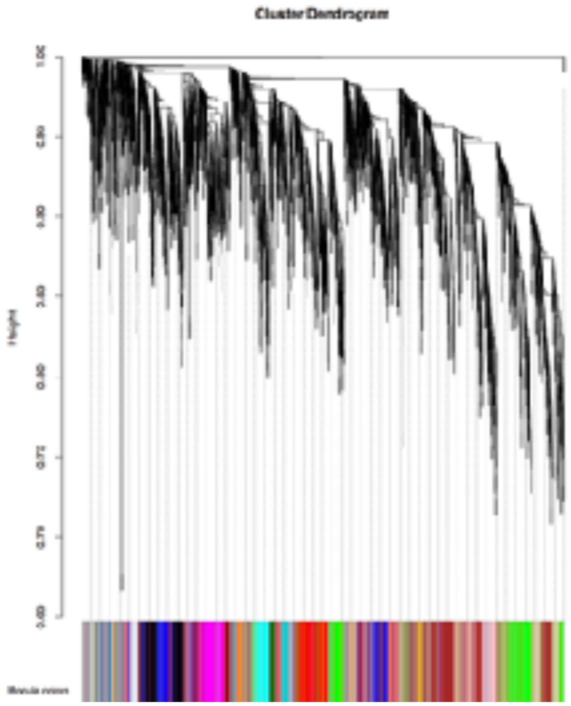

1B: BPA NETWORK DENDROGRAM

1C: LOW DOSE BPA NETWORK DENDROGRAM
