# Supplementary figures and images for: Weighted Gene Correlation Network Analysis (WGCNA) Reveals Novel Transcription Factors Associated With Bisphenol A Dose-Response
Source: Front Genet. 2018 Nov 12;9:508. doi: 10.3389/fgene.2018.00508 (PMC6240694; doi:10.3389/fgene.2018.00508)

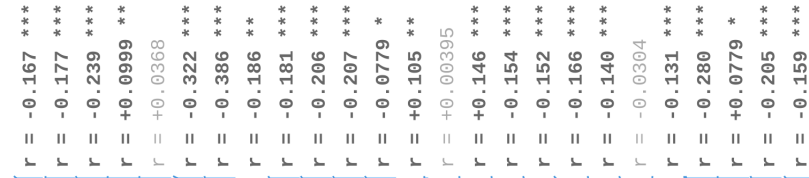

Supplement: FIGURE S2 — Methylation pattern for FIZ1 in BRCA TCGA data set. [file Data_Sheet_2.PDF]
